# Ligation of the Left Subclavian Arteries (Third Division) and the Transversalis Colli and Suprascalpular, with Cocaine Anæsthesia

**Published:** 1896-01

**Authors:** John A. Wyeth

**Affiliations:** New York


### Southern Medical Record.

A MONTHLY JOURNAL OF MEDICINE AND SURGERY.

VOL. XXVI. ATLANTA, GA., JANUARY, 1896. No. 1.

### Original Articles.

### LIGATION OF THE LEFT SUBCLAVIAN ARTERIES (THIRD DIVISION) AND THE TRANSVERSALIS COLLI AND SUPRASCAPULAR, WITH COCAINE ANÆSTHESIA. SUBSEQUENT BLOODLESS AMPUTATION AT THE SHOULDER JOINT.*

BY JOHN A. WYETH, NEW YORK.

John Simon, aged 31, by occupation a groceryman, with no personal or family history which throws any light on the case, came under my care October 2, 1895, in the New York Polyclinic Medical School and Hospital.

In 1889, he had received an injury of the left arm near the shoulder joint, which he considered a fracture, from which he recovered with some stiffness of the joint. He noticed nothing of interest beyond this until 1893, when, in descending from the deck of a ship, he caught hold of a rope to prevent himself from falling and severely strained this same arm and remembers having heard a ripping sound, followed by great pain. The arm has never been right since. Four or five months after this injury, he noticed it began to swell. It was not exceedingly painful, except on sudden motion.

When I saw him first, on October 2, 1895, there was a large, round swelling, which occupied the upper end of the humerus, the deltoid region, and extended somewhat over the

*Patient exhibited before the Surgical Section of the New York Academy of Medicine, [illegible]ember 9, 1895

end of the clavicle and scapula. On October 5, under chloroform anæsthesia, I made an exploratory incision for the purpose of getting a specimen for microscopical examination. Cutting directly down through the deltoid muscle, I came immediately into a large, vascular osteosarcoma, which had hollowed out the medullary canal and substance of the head of the humerus, into which I passed two or three fingers very readily. The bone gave way with a peculiar crackling sound, like the breaking of an eggshell and was followed by profuse hæmorrhage, which could only be arrested after rapidly packing the cavity with at least three yards of sterilized gauze; over this, an Esmarch elastic bandage was carried. This hæmorrhage greatly weakened the patient, and, by October 9, I became alarmed about his condition of anæmia, and injected into the right median cephalic vein a pint and a half of hot saline solution, one drachm of salt to a quart of water. This was followed by a rapid improvement in the heart action, fuller pulse, better appetite and increasing strength. There was, however, considerable hæmorrhage whenever the packing in the tumor was changed; so much so, that on October 15, in order to control this bleeding and to dispense with the packing, which was so tight that it caused him a great deal of suffering, I tied the left transversalis colli and suprascapular and the left subclavian arteries in the third surgical division. The operation was not easy of accomplishment by reason of the great swelling of the shoulder, which lifted the clavicle fully an inch higher than the normal position and prevented the manipulation of the parts, displacing the clavicle and shoulder on account of the great pain. The patient could not tolerate any pressure upon the shoulder. I was afraid to administer ether or chloroform on account of his exhausted condition, and, believing I could tie these arteries with local anæsthesia by the judicious employment of cocaine, it was done. In the entire procedure, twenty-five minims of a four per cent. solution were employed. The operation was quite tedious, and the subclavian artery, when finally exposed, was two and a half inches from the surface of the wound and deep below the clavicle, where it was caught up by an aneurism needle and tied. The patient lost practically no blood from the incision in the tumor after this operation.

On October 20, under ether anæsthesia, I made a rapid, bloodless amputation of the shoulder joint by the method which I first employed in doing this operation in 1889.

The operation consists in the insertion of two long, steel pins, about fourteen inches long and less than a quarter of an inch in diameter. The anterior one passes through the skin and part of the pectoralis major muscle, well back behind the level of the shoulder joint. Not more than one and a half inches of this pin should be concealed by the tissues it perforates. Over the spine of the scapula, well up behind the joint, the second pin is passed, taking hold of nothing but the integument, and, may be, a little of the surface of the trapezius muscle. Around the shoulder over the clavicle and scapula, and behind the pins, the points of which are shielded by bits of cork, strong rubber tubing is tightly wound four or five times, and then tied. The amputation can then be made at leisure, disarticulation completed, and the vessels taken up at will. The operation is entirely bloodless, and the tourniquet need not be removed until the procedure is completed. It is always advisable, however, to loosen the tourniquet before the final suturing in order to see that no important bleeding points have been overlooked.

I have performed three operations of a similar nature by this method, and a number of others have been done by competent surgeons, without hæmorrhage in any instance. It was the employment of this method in shoulder joint amputations, which led me to employ it in amputations at the hip joint, of which I have done seven with no hæmorrhage in any case, and only one death from the operations. The tourniquet remains in position until the operation is nearly completed.

In the present case, it being one of malignant tumor, and as the soft parts up to the level of the joint seemed to be involved, I did not endeavor to cover the joint surface with cutaneous flaps. It was my intention, as soon as the wound began to granulate and the bone to recover sufficiently, to infect it with some septic organism, preferably with streptococci erysipelatis, and on the 19th of November, an injection of five minims of pure culture of Fehleisen's coccus was done. They were repeated daily, or every other day, for two weeks, gradually increasing the dose by three minims until twenty-six minims were injected. So far, however, no regular erysipelas has been precipitated. He has had septic chills, with exacerbations of temperature following, which, on one occasion reached 40.7 degrees C. (105.5 degrees F.) These injections have been discontinued for the time being, and, despite the severe ordeal he has undergone, the patient has gained flesh and weight, and does not look like the same individual that came to me on the 2d of October.

The specimen which is here presented, shows an osteosarcoma of the upper end of the humerus. There is, as yet, no recurrence of the tumor.

---

A SEQUENCE OF OTORRHEA.—A rare case of disease of the middle ear, in which a resulting temporo-sphenoidal cerebral abscess discharged through the nose, is illustrated in the *Lancet* for June 29. The patient, a woman of thirty-eight, met with an accident in 1882, which excited an otorrhea, which was neglected. Eight years later she complained of a pain in the mastoid, which was opened, and on following up the track of the pus, a considerable amount was found between the dura and the bone and thoroughly evacuated. She made an apparently good recovery, but three years later returned to the hospital with pain in the head and pus oozing through a fistulous opening back of the ear. Optic neuritis could not be established. Facial paralysis set in rapidly. The condition became so serious that the mastoid was again opened and necrosed bone, with much pus, was removed. During the operation pus ran from the mouth in great abundance. The patient improved, though the pus continued to escape through the mouth and nostrils in small but constant amounts. A month after the second operation on the mastoid the patient died. The post-mortem examination showed a large amount of pus beneath the frontal lobe as well as above the same, and a track filled with pus extending *to the cribiform plate of the ethmoid.*—*Philadelphia Ledger.* If ethmoiditis anterior had been discovered in time, the woman's life might have been saved.—*St. Louis Medical and Surgical Journal.*

